# A unified dataset for pre-processed climate indicators weighted by gridded economic activity

**DOI:** 10.1038/s41597-024-03304-1

**Published:** 2024-05-24

**Authors:** Marco Gortan, Lorenzo Testa, Giorgio Fagiolo, Francesco Lamperti

**Affiliations:** 1 School of Finance, University of St. Gallen, St. Gallen, Switzerland; 2 Department of Statistics and Data Science, Carnegie Mellon University, Pittsburgh, PA USA; 3 Institute of Economics and L’EMbeDS, Sant’Anna School of Advanced Studies, Pisa, Italy; 4 RFF-CMCC European Institute on Economics and the Environment, Milan, Italy

**Keywords:** Climate change, Climate change, Economics

## Abstract

Although high-resolution gridded climate variables are provided by multiple sources, the need for country and region-specific climate data *weighted* by indicators of economic activity is becoming increasingly common in environmental and economic research. We process available information from different climate data sources to provide spatially aggregated data with global coverage for both countries (GADM0 resolution) and regions (GADM1 resolution) and for a variety of climate indicators (total precipitations, average temperatures, average SPEI). We weigh gridded climate data by population density, night-time light intensity, cropland, and concurrent population count – all proxies of economic activity – before aggregation. Climate variables are measured daily, monthly, and annually, covering (depending on the data source) a time window from 1900 (at the earliest) to 2023. We pipeline all the preprocessing procedures in a unified framework, and we validate our data through a systematic comparison with those employed in leading climate impact studies.

## Background & Summary

Climate change and weather events have been shown to adversely affect a wide spectrum of natural and socio-economic activities^1,2^. A blossoming body of literature reports evidence of significant and non-linear impacts on agricultural^3^ and economic production^4–6^, conflict^7^, income inequality^8^, mortality^9^, energy consumption^10^, and the list is far from being conclusive. Most of these studies test the presence of a significant statistical association between climate variables and socio-economic indicators, adopting either cross-section or panel-data approaches^11,12^.

One common challenge is that weather data are typically available at a much finer spatiotemporal resolution than socio-economic variables. While indicators such as industrial production, GDP, employment, and fatalities are typically collected annually – at region or country breakdowns – temperatures, precipitations, and other weather variables are instead available at gridded levels and hourly or daily frequency. Hence, the common approach requires weather-related variables to be aggregated to match lower temporal frequencies and the geographical boundaries of administrative units.

This process is not straightforward and often requires the use of weights proxying the geographical distribution of economic activities. Indeed, when studying the impact of climatic conditions and weather events on the economy, it is crucial to account for the different exposure of socio-economic activities within an administrative region. For example, average temperatures in the Mojave Desert (California, US) during the summer may be considerably higher than in Los Angeles (California, US), but the size of economic activities in the two locations is not even comparable. Indeed, one may easily argue that labor productivity in California is much more affected by temperatures in Los Angeles than in desert areas. Thus, a simple aggregation of climate data that does not account for the geography of socio-economic activities could introduce a bias in the evaluation of climate impacts, especially when the variability across administrative regions is central to the identification of the effect^11,12^. Further, when a weather-related phenomenon occurs at the regional level, in response to averaged weather, the weighting scheme is crucial to reflect the relative overall importance of weather in different regions. For instance, weighting rainfall by the distance from coastline could help to predict the declaration of states of emergency^11^.

Spatially weighted data are increasingly employed in the literature exploring the impacts of climate change and weather events on socio-economic activities. For example, Burke *et al*.^4^, in a seminal study assessing the effect of global warming on the dynamics of economic production, employ population-weighted temperatures and precipitations to measure gradual climate change. Accordingly, a number of studies have been relying on Burke *et al*. dataset to explore the impact of climate on economic inequality and growth^8,13,14^. Furthermore, population weighting is not limited to the case of average temperatures and total precipitations, as it is increasingly employed for a variety of additional climate indicators, e.g., in the evaluation of heating and cooling degree days^15^.

However, replicating published studies using spatially weighted climate data is difficult, as the exact procedure employed to obtain weighted climate variables used for impact assessment is often unclear, under-discussed, or not reported at all in existing contributions. This poses a potential problem, as the way in which weighting is performed may depend on a number of different key factors and choices^16^. Among them, the sources of data used for the construction of weights, the adjustments employed to align gridded information to the borders of administrative regions, and the eventual use of a base year are all elements that can sensibly affect the construction of spatially weighted climate indicators. This also undermines exercises trying to employ existing datasets containing spatially-weighted climate variables (e.g., made available in online repositories as supplementary material of published papers) in further studies or analyses. Indeed, in the absence of clear guidelines and documentation, it becomes very hard to build homogenized datasets covering different sets of countries or regions and longer time series (i.e., more recent years).

Here, we argue that the lack of a harmonized, documented, cross-validated, and open-access source for climate variables that are spatially weighted by economic activity hinders a rigorous and robust estimation of the social and economic impacts of climate change. This may partly explain why unweighted climate indicators are still employed in several studies. For example, in their main model specifications, Kotz *et al*.^6^ construct a number of indicators proxying the yearly distribution of rainfall within national and subnational regions without accounting for the spatial distribution of economic activities and use such indicators to show the adverse impact of precipitation extremes on economic growth – they adopt a specification with population-weighted variables in their supplementary material. Furthermore, spatially unweighted climate data are also employed in the emergent macro-econometric literature on climate impacts^17–20^.

In this paper, we try to close this gap by introducing a unified source of data that pipelines the preprocessing and weighting procedures of gridded climate data into a documented, intuitive, and open-access interface. The dataset allows researchers to get ready-to-use climate variables aggregated at national and sub-national levels, with global coverage over the period 1900–2023. Moreover, we provide a user-friendly dashboard to explore and download key climate variables under customizable weighting schemes, temporal frequency, timeframe, administrative level, and file format.

Our dataset is intended to support the climate impact assessment community, which is constantly enlarging and increasingly opening to scientists and researchers who aim to work with datasets compiled at the administrative level (e.g., economists and public policy scholars). Indeed, by offering a unified and harmonized access to a wealth of publicly available yet dispersed and unweighted climate and weather indicators, we aim to improve the replicability of impact assessment studies, increase the transparency of data management practices, and incentivize the community to test the robustness of estimates to the choice of data sources and aggregation strategies.

## Methods

**Fig. 1:**
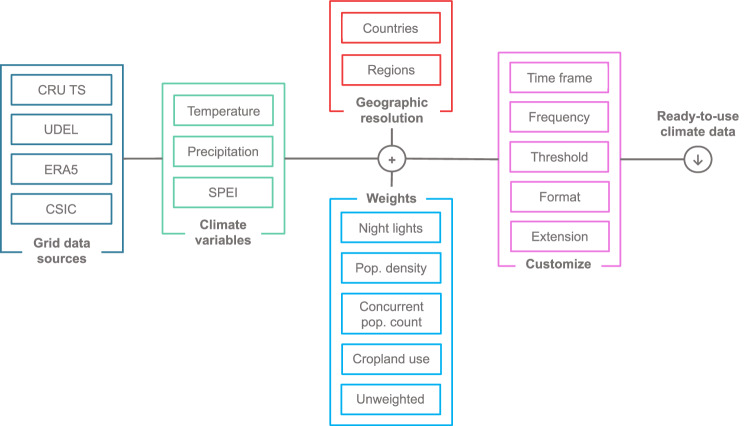
The *Weighted Climate Dataset* workflow. Users can combine gridded climate variables, gridded indicators of economic activity, and administrative boundaries to achieve regional climate variables *weighted* by economic activity.

The logical steps behind the construction of our dataset are illustrated in Fig. 1. We combine different gridded climate variables from multiple open-access sources, gridded indicators of spatial socio-economic activity, and administrative boundaries at different levels of resolution. The main objective is to obtain climate data that are weighted by socio-economic indicators according to different strategies that are customizable by the user. To achieve this, our procedure follows three key steps:

- **Selection**: In the first step, we choose (i) a specific set of gridded climate variables of interest, (ii) the desired geographical resolution, and (iii) a gridded economic activity indicator for constructing the aggregation weights.
- **Computation of weights**: Next, we integrate the selected information to derive a gridded weighted version of each climate variable. This process ensures that the socio-economic indicators are appropriately considered in the analysis.
- **Aggregation**: Finally, we aggregate the gridded weighted observations across the regions defined by the chosen geographical resolution. This step allows us to obtain a comprehensive view of climate data at the desired level of granularity.

An interactive interface enables users to explore the dataset, customize the aggregation process and the download format. They can modify parameters such as the base year for constructing weights, the frequency of climate data (i.e., daily, monthly, yearly), and the time span of interest. Additionally, users can access specific information in the dataset tailoring it to their end-use requirements.

### Gridded variables and administrative boundaries

The core of the *Weighted Climate Dataset* rests on two groups of gridded variables: climate variables and indicators of economic activity. These variables, together with administrative boundaries, serve as the fundamental components of our dataset. Table 1 shows all the sources of data we exploit in our work.

**Table 1:** Summary of the main features of the employed data sources.

Source	Reanalysis	Variables	Coverage period	Frequency	Resolution	Version
CRU TS^21^	No	Temperature, precipitation	1901–2022	Monthly	0.5°	4.07
CSIC^22^	No	SPEI 1-month	1901–2020	Monthly	0.5°	2.7
ERA5^37^	Yes	Temperature, precipitation	1940–2023	Daily	0.25°	5
UDEL^24^	No	Temperature, precipitation	1900–2017	Monthly	0.5°	5.01
GPW^30^		Population density	2000, 2005, 2010, 2015	Yearly	0.25°	4
Li *et al*.^31^		Night-time light intensity	2000, 2005, 2010, 2015	Yearly	$0.008{\bar{3}}^{\circ }$	7
HYDE^32^		Cropland	2000, 2005, 2010, 2015	Yearly	$0.08{\bar{3}}^{\circ }$	3.2
GPW^30^		Population count	2020	Yearly	0.25°	4
HYDE^32^		Population count	1900–2010	10 years	$0.08{\bar{3}}^{\circ }$	3.2
GADM^33^		Administrative boundaries				4.1

#### Climate data

We leverage raw gridded climate data from four sources that are routinely used in climate impact studies: Climate Research Unit Time-Series^21^ (CRU TS v4.07, available from 1901 until 2022), Consejo Superior de Investigaciones Científicas^22^ (CSIC v2.7, 1901–2020), ECMWF Reanalysis v5^23^ (ERA5, 1940–2023), and University of Delaware^24^ (UDEL v5.01, 1900–2017). CRU TS, UDEL, and CSIC provide data at the grid resolution of 0.5° × 0.5°, while data from ERA5 feature a finer resolution (0.25° × 0.25°). Each source offers *monthly* records for two climate indicators, namely average temperatures (measured in Celsius degrees, *C*) and total precipitations (in millimeters, mm), with the exception of CSIC, which provides monthly records for a third climate variable, the Standardized Precipitation-Evapotranspiration Index^25^, also known as SPEI (unit free). In addition to monthly data, ERA5 also provides records at the temporal resolution of *hours*, which we aggregate to obtain *daily* values.

CRU TS employs raw data from an extensive network of weather stations, computes monthly climate anomalies, and interpolates them using angular-distance weighting^21^ (ADW). ADW is employed to account for the varying area represented by each grid cell on a spherical Earth, in particular by considering the cosine of the latitude of each grid cell. The cosine of the latitude serves as a measure of the change in grid cell area with respect to latitude. Cells near the equator have larger areas as compared to those near the poles, where cells are smaller.

CSIC leverages CRU TS data to provide the SPEI, a drought index that combines information from both precipitation and evapotranspiration to assess the severity and duration of drought conditions. It is a standardized version of the widely used Palmer Drought Severity Index (PDSI) that takes into account the effects of both precipitation and temperature on water availability. Given its multi-scalar nature, it is able to differentiate among different types of drought; we currently propose the 1-month level of aggregation, focusing on changes in headwater levels.

ERA5 climate data set uses data from radiosondes, which are battery-powered telemetry instruments carried into the atmosphere by weather balloons to measure various atmospheric parameters, including temperature, wind, and humidity profiles. The information collected by radiosondes is transmitted back to the ground via radio signals and is assimilated by ERA5 along with other observations, such as satellite and surface-based measurements, using numerical weather models, in order to provide a comprehensive picture of the Earth’s climate system^23^. It is the only *reanalysis* source, as it integrates climate models with past observations to provide (i) consistent values over time and (ii) more accurate estimates in the grids not covered by measurement stations.

Finally, UDEL provides gridded estimates mainly based on station records compiled from several publicly available sources (e.g., Global Historical Climatology Network dataset^26^, Global Historical Climatology Network Monthly dataset^27^, the Daily Global Historical Climatology Network archive^28^). Interpolation is performed with Shepard spatial-interpolation algorithm^29^, modified for use over Earth’s near-spherical surface.

#### Socio-economic data

We use gridded socio-economic data to gauge information on the spatial distribution of economic and human-based activities. In particular, three distinct indicators are used as weights for the spatial aggregation of climate data into administrative units. The first proxy is population density, available from Columbia University’s Gridded Population of the World v4 (GPWv4)^30^, measured at 0.25° and 0.5° spatial resolutions. The climate econometrics literature has largely employed population density as an indicator of economic activity proxying local exposure to weather conditions^4,11,12^. Note that population density is measured with respect to the land area of each grid. Thus, in our aggregation strategy, we employ the product between the population density and the area of the associated grid to account for population size properly.

**Fig. 2:**
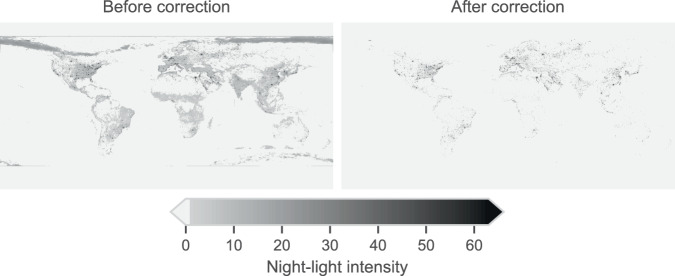
Correction of auroras and other noise sources in the night-time light data for the year 2015. The left plot shows night-time light data *before* correction; the right plot shows the same data *after* correction, which consists of setting to 0 the values in the grids whose digital number values are less than 30.

A second, alternative indicator of economic activity that we include in our dataset is night-time light data^31^. Records in night-time light data are the digital number (DN) values, a standard measure of the brightness of a pixel in a digital image ranging from 0 to 63. These data are originally available at a 30 arc-second spatial resolution ($0.008{\bar{3}}^{\circ }$). To match this finer resolution with the coarser resolutions of our gridded climate data, we compute the mean of the values of the cells in the 0.25° and 0.5° grids. We aggregate by first taking the mean of 900 (30 × 30) and 3600 (60 × 60) most upper-left cells in our coordinate system to produce a single grid at a resolution of, respectively, 0.25° and 0.5°. We then iterate this procedure with the adjacent blocks of cells to obtain all the gridded values of the night-time light data for the coarser resolution. We note that the harmonized VIIRS-DMSP tif file (especially for the year 2015) presented noise from auroras and other temporary effects (e.g., boat lights and fires) – see Fig. 2, left panel, where we show the aggregation for the year 2015. Therefore, as suggested by Li *et al*.^31^, we set to 0 the values in the grids whose DN values are less than 30 before aggregating. Figure 2, right panel, shows the result of this correction for the year 2015.

We also include a third proxy for economic activity in our dataset – cropland. Data on cropland are available from the History Database of the Global Environment (HYDE)^32^, version 3.2, and measure the area of the arable land and the permanent crops within each cell, in square kilometers. These data are recorded at a spatial resolution of 5 arc-minutes ($0.08{\bar{3}}^{\circ }$). To match the finer resolution of these grids with the coarser resolutions of our gridded climate data, we perform the same aggregation procedure as for the night-time light data. We notice that weighting climate variables by crop areas may be a relevant choice to reflect the impact of climate change in regions where crops are grown.

We allow weighting by population, night-time light, and cropland using the base years 2000, 2005, 2010, and 2015. Moreover, the dataset contains aggregated climate data which have not been weighted by any spatial economic indicator, but only by the area of each grid cell. This option is referred to as *unweighted*. Finally, we provide a different weighting strategy that we refer to as *concurrent*, where we weigh climate variables using the population count measured at the beginning of the *zero-to-nine* decade of reference, to provide an integrated dynamic weight. For example, temperatures in 1907 are weighted using population data in 1900. We exploit population count from HYDE^32^, version 3.2, for the decadal years from 1900 to 2010, and GPW, for the decadal year 2020, as HYDE (v3.2) population data are available until 2017. Note that HYDE adopts the United Nations World Populations Prospects (UN-WPP) as the basis for the post-1950 estimates. Therefore, we employ the *UN WPP-Adjusted Population Count* from GPW in order to make the two sources consistent.

#### Administrative boundaries

We employ two levels of geographical resolution from the Database of Global Administrative Areas^33^ (GADM). While the first level (GADM0) has a coarser resolution and replicates country boundaries, the second level (GADM1) is sub-national and consists of the largest administrative area included within national countries (e.g., states for the US, regions for Italy, etc.). In our work, we used GADM version 4.1 released on July 16, 2022.

### Weighting and aggregation strategy

Raw grid data require to be aggregated to match administrative areas, for which many other socio-economic indicators are usually available. The general weighting scheme is the following:

1 $${y}_{i,t,w,T}=\frac{{\sum }_{j\in {J}_{i}}{a}_{j}{f}_{i,j}{w}_{j,T}{x}_{i,t}}{{\sum }_{j\in {J}_{i}}{a}_{j}{f}_{i,j}{w}_{j,T}}$$

where *y*_*i,t,w,T*_ is the value of the climate variable *y* in the geographical unit *i* (at a specified GADM resolution) at time *t* weighted by proxy *w* measured in base year *T* ∈ {2000, 2005, 2010, 2015}; *J*_*i*_ is the set of grids intersecting the geographic unit *i*; *f*_*i, j*_ is the fraction of grid *j* which intersects the geographic unit *i*; *a*_*j*_ is the area of the grid *j*; *x*_*i, t*_ is the raw grid climate variable. In all but the *concurrent* aggregation scheme, in line with the prevailing practice in the literature, the base year *T* is fixed ex-ante and does not vary with *t*^4,12^. Of course, for the unweighted aggregation, *w*_*j, t*_ = 1 for any *j* and *T*. When applying the *cropland* and *concurrent* weights, we set *a*_*j*_ = 1, since the two measures do not need any adjustments for the area of the grid. Finally, in the *concurrent* aggregation, we have *T* = *h*(*t*), where *h* is a function taking the year of the date *t* and returning its decade-floor. For example, *h*(1948) = 1940.

**Fig. 3:**
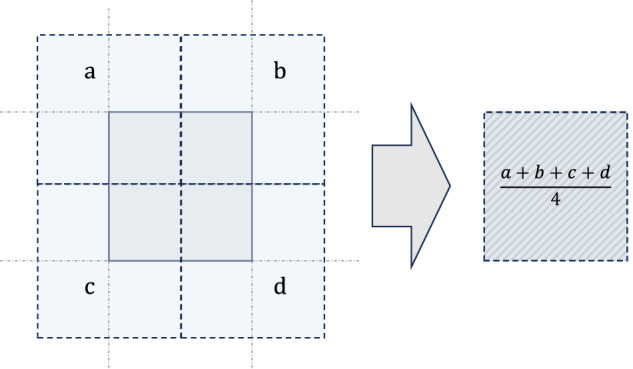
Stylized illustration of the bilinear interpolation when the population density, night-time light, cropland, and concurrent population count grids are used to weigh ERA5 climate variables. The extent of the weighting grids slightly differs from the extent of the ERA5 climate variables grids, both in longitude and latitude, resulting in a difference of 0.125° in both directions, exactly half of the spatial resolution of the ERA5 data. Since our weighting procedure requires weighting and variable grids to overlap, we resample the weighting grids, filling the values with a simple average of the values of the intersecting grids.

We notice that grid resolutions may vary across data sources. The NetCDF file retrievable from ERA5 is made up of a 721 × 1440 grid, with extremities (180.125°W, 179.875°E, 90.125°S, 90.125°N), and a 15 arc-minute spatial resolution. The gridded files of the weights feature instead a 720 × 1440 grid, with extremities (180°W, 180°E, 90°S, 90°N). To make the weighting and climate variables of ERA5 consistent, we resampled the values of the weight grids with a simple bilinear interpolation. The logic behind such a procedure is sketched in Fig. 3, where the stylized grids of two sources are displayed. This procedure is applied whenever we weigh climate variables from ERA5 with population density, night-time light, cropland, and concurrent population count grid files.

Sources of both climate and socio-economic data sporadically present missing values. We deal with this issue conservatively: when we are not able to properly weigh the climate variables (for example because the weights are all 0, or because climate sources do not provide data for cells in a specific geographical unit), we do not impute values, and leave NAs instead.

**Fig. 4:**
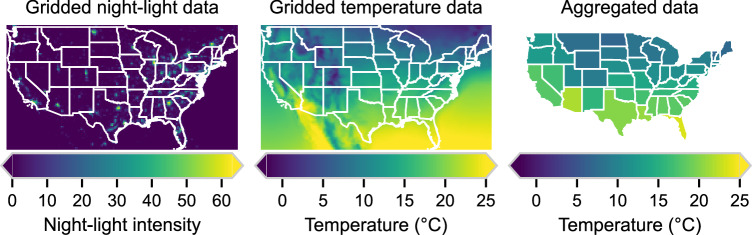
Example of climate data weighting for the US. The left panel shows raw gridded night-light data in 2015. The middle panel displays raw gridded temperature data in 2015. Finally, the right panel shows, for the year 2015, temperatures aggregated at the GADM1 administrative level *weighted by* night-lights in 2015.

As an example of the aggregation strategy, Fig. 4 shows three panels. The left and center panels display raw gridded data for night-time light intensity in 2015, and ERA5 average annual temperatures in 2015 for the contiguous US, respectively. Night-time lights are chosen as the weighting variable in this example. Figure 4, right panel, displays the resulting aggregation at GADM1 resolution and illustrates the output that users can retrieve from our dataset.

## Data Records

Data are available at Figshare^34^. The repository contains datasets relating to 216 different combinations of geographical resolution (GADM0, GADM1), climate variable (temperature, precipitation, SPEI), climate data source (CRU TS, UDEL, ERA5, CSIC), weighting variable (unweighted, population density, night-time light, cropland, concurrent population), time resolution (daily, monthly), and weighting base year (2000, 2005, 2010, 2015). Each combination is stored in a separate file at Figshare^34^, saved in csv format. These are organized in a folder with two layers, where the first corresponds to a choice of geographical resolutions, and the second discriminates among the climate variables. Each dataset is organized in *wide* format, where the first column refers to the month (or the day), and the remaining columns, which are identified by the GADM code of the geographical units, contain the values of the weighted climate variable.

## Technical Validation

In this section, we validate our dataset against those employed in two influential climate econometric exercises: Kotz *et al*.^6^ and Burke *at al*.^4^. We evaluate the agreement between our weighting procedures and those obtained by these two studies, with the aim of supporting the reliability and effectiveness of our approach.

In order to conduct a proper validation exercise, we first align our data sources with the exact versions employed by the two targeted studies, which of course have been employing older versions for both climate and economic activity datasets. This allows us to validate the accuracy and robustness of our data processing pipelines and methods, and to ensure a fair and reliable assessment of the quality and consistency of our estimates.

More precisely, Burke *et al*.^4^ exploit UDEL v3.01 for precipitation and temperature data, and v3 of the GPW 0.50° gridded population data in 2000. Population is used as the weighting variable and, although the authors do not specify the source and version of the national administrative boundaries they use, their shape files are publicly available. Conversely, in their main specification, Kotz *et al*.^6^ use 0.25° gridded ERA5 precipitation and temperature data, do not weigh climate data with any indicator of economic activity, and employ GADM1 v3.6 for the spatial aggregation.

**Fig. 5:**
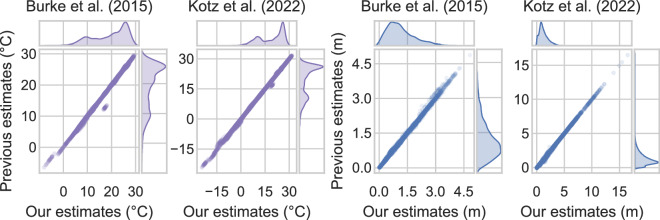
Comparison of weighted and/or aggregated temperature and precipitation variables in our datasets against data used in Burke *et al*. (countries) and Kotz *et al*. (sub-national regions). Average yearly temperature is expressed in degrees Celsius while annual total precipitations are in meters. Values on the main diagonal indicate very similar estimates.

Results of our comparative analysis are reported in Fig. 5. The figure includes four scatterplots, each representing the relationship between our estimates and those used in the original studies for both temperature and rainfall (SPEI is not used in either of the two mentioned works). Intuitively, points aligning on the main diagonal of the scatterplots indicate agreement and reflect the similarity between the estimates.

It is important to note that the data shown in Fig. 5 encompass all the years analyzed in the original studies. Notably, a substantial majority of our estimates exhibit a high degree of correspondence with the weighted and/or aggregated data employed by previous authors. This indicates a strong level of agreement between our results and those of previous studies, corroborating the quality and reliability of the methods employed to build our dataset. However, there also emerge some minor discrepancies that are worth pointing out. In particular, the first panel on the left highlights two main sources of disagreement between the estimates of Burke *et al*. and ours. The first one, on the bottom left (where both temperatures are negative), regards Greenland. In this case, the estimates of Burke *et al*. are higher than ours. The second one, where the estimates of Burke *et al*. are instead slightly smaller than ours, concerns Bhutan. These discrepancies are mainly due to the weighting scheme, and in particular to the fact that population density is highly concentrated in a few regions of Greenland and Bhutan.

**Fig. 6:**
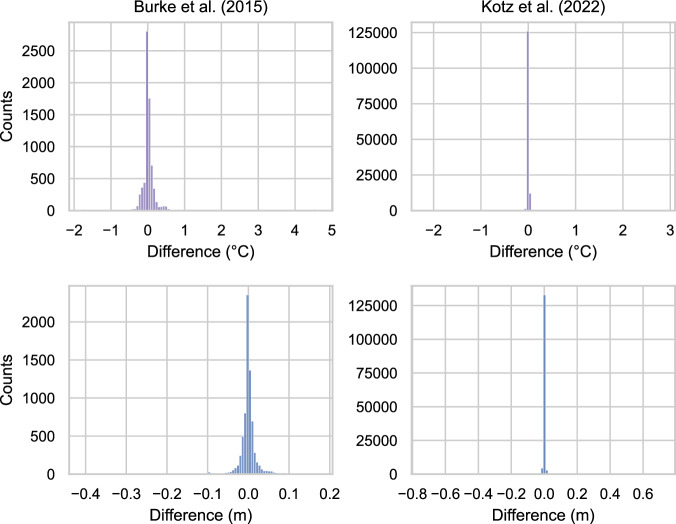
Comparison of weighted and/or aggregated temperature and precipitation variables in our datasets against data used in Burke *et al*. (countries) and Kotz *et al*. (sub-national regions). Average yearly temperature is expressed in degrees Celsius while annual total precipitations are in meters. Each histogram displays the difference between our and other authors’ estimates.

Similarly, Fig. 6 shows the same information presented in Fig. 5 in a different way. In particular, each histogram represents the distribution of the difference between our estimates and those of the other authors. Clearly, the histograms peak at 0, suggesting that our estimates are very similar to those performed by other studies.

## Usage Notes

In addition to the repository data, we have also made these data available in the *Weighted Climate Dataset* dashboard, which can be accessed at https://weightedclimatedata.streamlit.app.

## Data Availability

Python code running the *Weighted Climate Dataset* dashboard and scripts for aggregating data are available at https://github.com/CoMoS-SA/climaterepo. The *Weighted Climate Dataset* leverages Streamlit. We employed R^35^ to process the data, exploiting package exactextractr^36^ for the weighted aggregations.
